# Trajectories of Coping With Persistent Smell and Taste Dysfunction After a Covid‐19 Infection—A Qualitative Interview Study

**DOI:** 10.1111/jan.16601

**Published:** 2024-11-07

**Authors:** Pernilla Sandvik, Nicklas Neuman, Maja Flodin, Elin Lövestam

**Affiliations:** ^1^ Department of Food Studies, Nutrition and Dietetics Uppsala University Uppsala Sweden

**Keywords:** anosmia, olfactory training, parosmia, treatment

## Abstract

**Aim:**

To explore trajectories of understanding and managing persistent chemosensory dysfunction after COVID‐19 in patients undergoing clinical treatment.

**Design:**

A descriptive qualitative interview study with a realist approach.

**Method:**

Data were collected in Sweden, from August 2022 to March 2023 through semi‐structured interviews with 30 patients undergoing treatment for long‐lasting smell and taste dysfunction resulting from COVID‐19. Thematic analysis was applied in a process that involved continuous discussions and refinement of codes into themes and subthemes.

**Results:**

Three main themes were identified: (1) Understanding the sensory alterations, which includes subthemes of Searching for validation and Seeking remedies; (2) Practical coping strategies, encompassing Adapted eating and Managing olfactory training and (3) Navigating the emotional landscape, featuring Self‐persuasion and suppression and The ambivalence of acceptance. These themes highlight the cyclic and fluid nature of coping with the dysfunction, reflecting a dynamic process of adaptation. Despite treatment options like olfactory training, participants frequently experienced frustration due to limited perceived improvement and the psychological toll of managing their condition.

**Conclusion:**

The study highlights the complex and personal nature of coping with long‐term smell and taste dysfunction post‐COVID‐19. Coping strategies varied widely and evolved over time, reflecting a dynamic process of adaptation. The emotional and psychological impact was profound, underscoring the need for comprehensive treatment approaches that include both physiological and psychological support. There is a need for more effective interventions and support mechanisms to improve the quality of life for those affected by these persistent symptoms.

**Implications:**

This study contributes to the understanding of COVID‐19's long‐term effects on smell and taste, highlighting the need for ongoing support from health care, further research into effective interventions, and a comprehensive approach to care for individuals affected by these conditions.

**Impact:**

This study addresses trajectories and challenges of coping with persistent chemosensory dysfunction. Findings emphasise the complexity of managing persistent chemosensory dysfunction and the critical role of personalised care. The study contributes to knowledge for nurses in understanding patients with chemosensory dysfunction.

**Reporting Method:**

This research adheres to the EQUATOR guidelines: Standards for reporting qualitative research (SRQR).

**Patient or Public Contribution:**

No patient or public contribution.

## Summary

- What does this paper contribute to the wider global clinical community?
  ○ Further knowledge about how the long‐term consequences of COVID‐19 continue to affect some people, here in the form of persistent chemosensory dysfunction.
  ○ An understanding of what type of support these patients need, particularly when the chemosensory dysfunction is persistent.
  ○ Based on the patient's experiences, we highlight the challenges and complexities of using olfactory training as a treatment, particularly in parosmia.

## 1 Introduction

Reductions and distortions in the sense of smell and taste (chemosensory dysfunction) are common to a range of illnesses and conditions, with a variety of underlying causes, symptom severities, durations and available treatments (Spielman 1998). In the spring of 2020, a new illness was added to the list: COVID‐19. While initial reports mostly focused on symptoms like dry cough and fever, it soon became apparent that sudden loss of taste and smell was a common characteristic of the disease (Parma et al. 2020). The symptoms not only included a decrease in smell sensitivity, such as anosmia and hyposmia, but also qualitative alterations in smell perception, like parosmia and phantosmia (Parma et al. 2020). Additionally, the virus affected taste by causing both a reduction in taste sensitivity (ageusia and hypogeusia) and qualitative changes in taste perception (dysgeusia and phantogeusia) (Parma et al. 2020; Vargas‐Gandica et al. 2020). The exact prevalence estimates vary, but numbers as high as 80% for some form of chemosensory symptom have been reported (Santos et al. 2021).

As the pandemic progressed, it became clear that COVID‐19‐induced chemosensory dysfunction stood out markedly from similar symptoms caused by other respiratory infections. This was partly about prevalence, but even more about its severity and remarkable persistence. Estimates based on international literature suggest that approximately 5% of those affected still experienced some form of dysfunction after 180 days (Tan et al. 2022). Long‐term follow‐ups are few, but the ones that exist demonstrate that a small group still have symptoms up to 24 months after onset (Melanie et al. 2024). Various hypotheses have been suggested about the precise physiological reasons, and while some have received more support than others, no consensus exists (Butowt, Bilinska, and von Bartheld 2023), and there is yet no answer to the question of whether some will be affected for the rest of their lives.

## 2 Background

Individuals affected by chemosensory dysfunction caused by COVID‐19 often describe struggling with unpleasant, distorted odours (parosmia). Words used to characterise these smells include sewage, chemical, acrid, metallic and burnt (Burges Watson et al. 2018, 2021; Parker et al. 2022). The foods affected vary between individuals but commonly mentioned foods are coffee, meat products and various types of onions (Parker et al. 2022). Those affected find it difficult to describe their experiences of these new odours but associate them with things like strong chemicals, ash and even faeces (Parker et al. 2022). For many, distorted smells can be so intense that they induce gagging or even vomiting. Furthermore, some report a specific ‘COVID smell’, described as a never‐before‐experienced and very unpleasant odour (Burges Watson et al. 2018, 2021).

With regards to possible treatments, reviews discuss the potential benefits of systematic olfactory training and corticosteroids but conclude a near absence of evidence to inform treatment recommendations, particularly for qualitative olfactory dysfunction (Helman et al. 2022; Liu et al. 2023). Although developed for olfactory loss (anosmia and hyposmia) caused by other illnesses and conditions, systematic olfactory training is the first‐choice treatment for symptoms caused by COVID‐19 as well, yet randomised controlled studies are needed to optimise the treatment (Pieniak et al. 2022). In addition, a couple of studies have specifically described olfactory training as a possibly effective treatment method for COVID‐19‐induced parosmia (Altundag, Yilmaz, and Kesimli 2022; Liu et al. 2021). All in all, however, the evidence in favour of meaningful improvements from the currently offered treatment options is limited.

This paper addresses the trajectory of having to cope with chemosensory dysfunction of varied types and severities, something that can be stressful and detrimental to the quality of life (Croy, Nordin, and Hummel 2014; Llana et al. 2023; Winter et al. 2023). The process of coping with distress has been widely theorised, with one influential example being Lazarus and Folkman's psychological stress and coping theory (Biggs, Brough, and Drummond 2017; Folkman and Lazarus 1980). According to this theory, individuals are constantly appraising stimuli within the environment, a process that generates emotions, and when stimuli are appraised as threatening, challenging or harmful it creates distress. As an example, having a favourite food or all foods suddenly tasting awful because of parosmia could be considered stressful. According to the theory, this would initiate coping strategies to manage emotions or attempts to address the stressor itself. Some concrete examples of this have been identified in a scoping review of food‐related experiences and behavioural responses to COVID‐19‐induced chemosensory dysfunction, these include avoidance of repulsive smells and tastes, and modifications of foods to make them more palatable or sensorily stimulating (Neuman et al. 2023).

Different needs may arise over time and coping strategies may need to be exchanged or adjusted. Yet, the review did not find any publications addressing the long‐term perspectives of living with and coping with these symptoms, nor were the strategies used from symptom onset to living with them for years explored (Neuman et al. 2023). Lastly, none of the identified studies in the above‐mentioned review focused on patients who have experienced clinical treatment for their problems (Neuman et al. 2023). Therefore, there is a need for an increased understanding of how patients manage chemosensory dysfunction, especially those affected as a result of a COVID‐19 infection.

## 3 The Study

### 3.1 Aim

To describe trajectories of understanding and managing persistent chemosensory dysfunction after COVID‐19 in patients undergoing clinical treatment.

## 4 Method

### 4.1 Design

This is a descriptive qualitative interview study based on a realist approach. The descriptive approach is particularly well suited for capturing participants' detailed experiences in straightforward, everyday terms, emphasising the provision of an authentic and comprehensive summary of participants' narratives (Sandelowski 2000, 2010). Data were gathered from semi‐structured interviews (Brinkmann 2014) exploring COVID‐19 patients' experiences of, and strategies around, smell and taste changes in their everyday life with a particular, although non‐exclusive focus on food and meals. The interviews provided rich, detailed stories that captured the complexity and depth of participants' experiences. Data were analysed through realist thematic analysis (Braun and Clarke 2006), an approach that treats the data as evidence for real experiences and emotions, existing independently of our beliefs and discourses about them yet filtered through the lens of the participants' language and worldviews (Maxwell 2024). In other words, the approach assumes that participants' accounts are incomplete and fallible, but reflects an objective reality (e.g., their sensory perceptions and their emotional experiences) for researchers to approximate (Porter 2007).

### 4.2 Study Setting and Recruitment

Patients were recruited from a Swedish clinic dedicated to structured olfactory rehabilitation, established in autumn 2021 in response to a rapidly increased demand from the pandemic. During their consultation with the clinic, patients received an overview of the study from the lead nurse and were handed a concise informational pamphlet, which included a QR code for further details. Scanning the QR code redirected patients to REDCap (Research Electronic Data Capture), a secure online platform for data storage. Here, individuals could consent to participate and submit their contact information. Subsequently, registrants were approached by either PS, NN or EL to schedule an interview at a convenient location and time.

### 4.3 Inclusion and Exclusion Criteria

Patients at the participating clinic experiencing chemosensory dysfunction caused by COVID‐19 were included. Eligibility for participation was limited to individuals over 18, fluent in Swedish, and without any reported eating‐related impairments such as significant allergies or mastication or swallowing difficulties.

### 4.4 Data Collection

The interviews were conducted by P.S., N.N. or E.L., in Swedish, at a location suggested by the participant such as their workplace, a calm café, in their home or by an online video meeting. They varied in length from 30 min to 2 h and were audio‐recorded. Data were collected between August 2022 and March 2023. The decision to stop was made after deliberation over meaning saturation (Hennink, Kaiser, and Marconi 2017) between P.S., N.N. and E.L. The process of reaching saturation included chunks of data that had gone through preliminary analyses during the interviewing process and the three interviewing researchers engaged in constant discussion about their impressions. When an agreement about saturation had been reached, a decision was made to close recruitment on March 31, 2023. Out of 31 individuals who expressed interest, 30 were interviewed (Table 1). The semi‐structured interviews were structured around a guide developed by the research team and clinic‐affiliated researchers. It was divided into three parts: (1) the symptoms, (2) everyday life and (3) the future. These covered treatment experiences and symptom progression, personal coping strategies and social interactions, daily dietary habits, social activities and the symptoms' impact on health and future healthcare needs (see Data S1). The guide was followed by the interviewers but with flexibility to ensure flow in the conversation, all topics and questions were, however, covered in each interview. Participants also completed a brief questionnaire detailing demographic‐ and symptom‐related information (see Data S2).

**TABLE 1:** Characteristics of the participants (*n* = 30).

Mean age in years (range)	42 (20–64)
Gender (*n*)	
Female	25
Male	5
Educational level (*n*)	
Secondary school	6
University	24
Occupation (*n*)	
Student	1
Employed	23
Retired	3
Parental leave	2
Sick leave	1
Household type (*n*)	
Single	6
With partner/children/roommates	24
Symptoms at debut/time of interview (*n* ^a^)	
Anosmia	26/6
Hyposmia	3/17
Parosmia/phantosmia	6/27
Ageusia	15/7
Hypogeusia	7/12
Dysgeusia/phantogeusia	6/23
Duration of symptoms in months, mean (range)	25 (9–36)

^a^ Multiple answers were possible.

### 4.5 Data Analysis

Transcriptions were carried out by students who used portions of the data for their theses (two BSc and one MSc), with one transcription completed by P.S. This allowed P.S., N.N. and E.L. to discuss the data at different stages while proceeding with the interview process. The transcriptions were based on the audio recording and included all verbal communication including pauses and emotional expressions. The transcriptions were kept in their original language (Swedish) during the whole analysis process.

The analysis was performed in two steps. First, while data collection was still ongoing, the research team reviewed transcriptions in segments, allowing for ongoing discussion and comparison of data. This initial analysis process facilitated a comprehensive understanding of the data, leading to a structured plan for further in‐depth analysis with separate focuses. Thus, a study focusing on disruptions of the participants' social lives has previously been published (Neuman et al. 2024) while the present study focuses on trajectories of coping with persistent smell and taste dysfunction.

In the second step, the realist thematic analysis (Braun and Clarke 2006) continued into a more systematic phase of coding. Here, relevant data extracts for the aim of the present paper were identified by P.S., saved as a txt file and imported into OpenCode 4.0.3, a tool for coding text‐based qualitative data (ICT Services and System Development and Division of Epidemiology and Global Health 2015). Data deemed relevant was related to participants talking about different stages of understanding and managing their symptoms, from early onset to present and thoughts about the future. The coding process was inductive, involving continuous interpretative interaction with the data. One code could vary in length, ranging from a few words to entire sentences. These codes were then grouped into blocks representing preliminary themes. During the coding process, specific excerpts and quotes from the participants' interviews were identified as exemplars—key pieces of data that clearly illustrated the identified themes and subthemes. The translation of the quotes to English was performed by the authors while the original citations in Swedish were kept in the manuscript together with the English translation during the whole writing process. These exemplars were selected to represent the broader patterns identified in the data, providing concrete examples to support and substantiate the thematic findings. The themes were subsequently discussed, revised and refined by the entire group of authors.

In the final part of the paper, the findings are discussed in relation to previous research as well as in relation to Lazarus and Folkman's psychological stress and coping theory (Biggs, Brough, and Drummond 2017; Folkman and Lazarus 1980). This theory is used to conceptualise and make sense of our findings. All authors participated in the final analysis and contributed to the writing of the findings.

### 4.6 Ethical Considerations

This study received approval from the Swedish Ethical Review Authority in May 2022 (No. 2022‐02353‐01). Crucially, the treatment providers, including the nurse, were unaware of who participated in the study, ensuring the independence of this study from the clinical treatment. This independence was also communicated to all participants. The data of each participant were assigned a unique identifier to ensure confidentiality. All collected data were securely stored in a cloud service managed by Uppsala University, with participant identifiers and contact information kept separately in REDCap. Transcription tasks were assigned to students working on their theses and were conducted within a secure segment of the cloud, adhering to strict confidentiality guidelines. In the results, we present the participants using pseudonymised names.

### 4.7 Rigour and Reflexivity

This study was committed to ensuring validity through a robust data collection and analysis process. Semi‐structured interviews enabled participants to share their experiences in detail, ensuring their perspectives were captured in depth. Two pilot interviews were conducted, reviewed and deemed satisfactory ultimately being included in the final analysis.

Accuracy was ensured through thorough documentation of the research process allowing for replication, consistency and reliability. By grounding our analysis in the participants' own words, we sought to enhance confidence in our interpretations (Porter 2007) by aligning the identified themes closely with the participants' perspectives and experiences. Authors thoroughly read the interviews multiple times, collaboratively discussing their interview experiences, preliminary analyses and suggested themes within the group. Discussions of our findings in the context of the existing empirical literature and theory further support the study's credibility. Transferability was considered by providing detailed descriptions of the study context, participant characteristics and findings allowing readers to assess the applicability in other contexts. Though conducted in a Swedish clinic, the participants' experiences are likely relevant elsewhere, enhancing the transferability of the findings. Member checking was not formally conducted. The study adhered to the Standards for Reporting Qualitative Research (O'Brien et al. 2014).

The interviewers are experienced qualitative researchers and represent diverse research fields, including sensory science, clinical dietetics and the sociology of food, providing a multidisciplinary perspective. Reflexivity was integral to our approach as the authors continuously discussed how their different backgrounds and pre‐understandings may have influenced the research process. This reflexive dialogue extended to how individual researchers approached interviews, such as the choice of follow‐up questions, and the various lenses through which they interpreted the data. The ambition was a deliberative, reflexive and transparent exchange of pre‐understandings and experiences so that the trustworthiness of the process could be maintained (Nowell et al. 2017). Importantly, following a realist paradigm, this process of reflexive deliberation means that we acknowledge biases and different perspectives, yet reject the assumption that they are equally valid (Maxwell 2024; Porter 2007). Instead, we assume that by critically examining these differences by discussing and testing them against data, we can reach a consensus that better represents the reality of the participants, and thus, consequently, provides nurses with more valid and actionable information.

## 5 Findings

**FIGURE 1:**
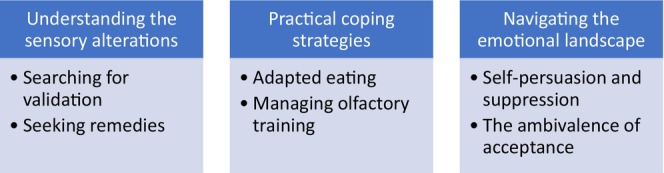
Themes and subthemes.

A sudden onset of smell and taste alterations was a commonly reported phenomenon among the participants, followed by a gradual improvement in sensitivity along with distortions. For some, these alterations occurred in conjunction with other COVID‐19‐related symptoms, and they described a wide span of experienced illness severity. For others, smell and taste dysfunction were the sole manifestation of the infection. Two participants, however, attributed their symptoms to the vaccine rather than the infection itself and one participant experienced the burden of both COVID‐19 symptoms and symptoms of early pregnancy such as nausea. The intensity of the taste and smell alterations varied among the individuals, influencing their respective strategies for managing the condition. Nevertheless, all participants experienced symptoms of sufficient severity to warrant seeking and receiving treatment at a specialised clinic. It was observed that the participants' strategies for managing the symptoms evolved. It is therefore essential to acknowledge the patients' coping with chemosensory dysfunction as a cyclic and fluid process. This underscores the deeply personal and non‐linear nature of their experiences, highlighting the interplay between different stages of coping and adaptation. The three themes described below represent different phases of this ongoing process (Figure 1). Although the process is here illustrated as phases, it is important to recognise the continuous nature and individually varied chronology of the experiences.

### 5.1 Understanding the Sensory Alterations

This theme illustrates how participants comprehend the nature and severity of their condition and determine their subsequent actions. It includes the subthemes (i) searching for validation and (ii) seeking remedies.

#### 5.1.1 Searching for Validation

The symptoms often manifested rapidly and unexpectedly, especially among patients who were affected in the early stages of the pandemic. A typical initial response among the participants was to attribute the sudden unpleasant taste or odour of food to spoilage, before realising that the changes were actually due to alterations in their own sensory perceptions.

So, I was drinking Coke and I was like, “God! This one's old” or I was like eating a bell pepper and threw it away and was like, “God, I bought a disgusting bell pepper.” And then when others started tasting it, they were like, “My God! This is not, what's wrong with you? This doesn't taste weird.” And then, damn, at that point I didn't know what this was. (Lisa, a woman in her late 20s, symptoms for 29 months).

Searching for information online and on social media emerged as a prevalent initial action upon recognising their symptoms. This approach served to confirm the legitimacy of their condition mainly by the existence of others with similar experiences. As Lisa put it, it felt supportive to know that ‘it's not just something psychological if everyone, if a lot of people have gotten this in exactly the same way then it's something … that should be possible to do something about’. However, accounts on social media of individuals recovering their sensory capabilities offered a paradoxical effect—instilling hope yet simultaneously causing dismay for those who had been symptomatic over extended periods. Moreover, although the groups offered a strategy for support at an early point of receiving the symptoms, the discussions seemed to reset with the arrival of newly affected individuals, perpetuating a cycle of repetitive questions.

And then new people come in too and you're like “Yes, so shall we start over?” It's like this “Oh. I've just lost. What can you do?” Such stupid questions. Sorry but people are so clueless. “What, what, what are safe foods for you?” You're like “Safe food? But we are all different. There are no safe foods. What are you talking about?” Just because someone can eat like vanilla then there are so many who can't eat vanilla. (Pia, a woman in her 50s, symptoms for 13 months).

Common to Lisa, Pia and others with similar stories, the symptoms were first managed by casting a wide net to understand what they were experiencing. It could be about asking others in their close social circles or searching online. While somewhat enlightening, it appeared to also come with the disadvantages of providing repetitive and unsatisfying information and, worse, instilling false hope.

#### 5.1.2 Seeking Remedies

In time, all participants eventually contacted healthcare due to their symptoms, receiving a range of treatments. In some cases, healthcare professionals conducted tests to exclude other conditions such as brain tumours, fungal infections of the tongue or nasal polyps, leaving some patients without clear diagnoses. A minority had the opportunity to consult with a dietitian, with both positive and negative experiences. Mona, whose symptoms debuted in October 2020, ‘couldn't find anything about it’ at the beginning. But then one of her colleagues suggested that she would go talk to the primary care centre.

So, then I did that and then [the doctor] called it parosmia, and then … there wasn't much more to it. But we, at least we did an MRI of the brain and such to make sure it wasn't anything else. (Mona, a woman in her late mid‐40s, symptoms for 24 months).

For individuals affected later during the pandemic, an increased volume of information became available. Testimonials, particularly concerning anosmia, were widely accessible on various media platforms, along with guidance on olfactory training. The general awareness among healthcare professionals increased as well, and olfactory training began to be recommended more frequently. Given the few treatment options available, some participants described themselves as soaking up all advice and ideas that they could find. Maria reflected on this.

[Y]ou do it because you read: “Try this, it might help.” You're so eager to get rid of it so you try it. And some are like, “Put garlic in your nose and sleep with it.” And you're like “Damn, that's gross, but I'll try, it might help.” And then, like people were making fun of it, to see how far a person is willing to go with this. And if you have these problems then you know, you're ready to do anything. (Maria, a woman in her late 20s, symptoms for 35 months).

Pia (woman in her 50s, symptoms for 13 months) reasoned that ‘there must be something more they have researched’, hoping for ‘something more like “But try cortisone” […] but now it's just this smell training. So, it's disappointing’. Echoing this longing for something new, participants described trying to be on top of any new recommendations, tips or tricks related to their condition. Yet another participant described how she had attended costly alternative treatments such as acupuncture and biomagnetism:

But then I also sat down and started calculating and thought, now I've spent over 25,000 [SEK, roughly €2,000] trying to get this started. Now I think I should let my body rest and be and then we'll see what happens. Because it's a lot of money, but it's also like this, if someone said “Rut, if you jump off a bridge then you will get this back,” then you would do it when you're most desperate. (Rut, a woman in her early 40s, symptoms for 36 months).

Maria's and Rut's quotes exemplify different ways of how participants were seeking remedies for their symptoms. Maria further stated that she would even prefer going completely anosmic than living with parosmia: ‘I hope you can kill the taste cells so that instead you don't feel any smell or taste at all…’ The fact that ‘you're ready to do anything’, as she put it, demonstrates that not only were patients involved in the medical treatment they finally received at the clinic but also experimented with complements in case something happened. The following theme deep‐dives into details of practical coping strategies, zooming in on both everyday adaptations of food and eating and specifics of the olfactory training.

### 5.2 Practical Coping Strategies

Over time, different actions were used to accommodate the situation and these also varied due to the manifestation of the symptoms, original preferences, routines and personality of the person. Two subthemes are included: (i) adapted eating and (ii) managing olfactory training.

#### 5.2.1 Adapted Eating

For some participants, eating had become substantially limited. Due to the few treatment options and the individual nature of their smell and taste dysfunction, they were also compelled to forge their paths in managing food intake. This process is illustrated by Birgit (a woman in her mid‐60s, symptoms for 18 months), describing how some things were impossible for her to eat, although ‘over time’, she said, ‘you also learn what, what you need to avoid’. While experimental strategies to manage unpleasant exposures were described, such as nose clips and face masks, the most common strategy in parosmia was to avoid all foods that triggered bad sensations. Penny (a woman in her mid‐20s, symptoms for 31 months) describes how it was worse in the beginning: ‘Yes, extremely difficult’, she told the interviewer, further explaining that ‘[I] don't even know what I was living on then. Like cold potatoes or cold pasta’.

An opposite strategy was active exposure to unpleasant smells or tastes, trying to endure them despite the discomfort. The hope was that this would lead to a quicker return to normal taste. Some described it as ‘eating training’ or ‘hardcore training’, meaning committed exposure to specific foods that tasted awful, with the hope that eventually they would get used to the new flavour. This strategy worked for some in the sense that the food became bearable to eat, although still not tasty. Eva elaborated on this strategy.

I've also, and especially lately, forced myself to eat things I know taste bad at least once a day, because, I also believe that's the way out of this, now when the smell training maybe isn't having much effect, I also have to start forcing my taste buds in some way to get used to these flavors […] The first reaction is to just want to spit it out uh, and you just take one bite, I mean if it tastes really, really bad then you don't eat any more of it. Uh, it's, well, what you have to do, even now. I know cucumber tastes terribly bad but I know that, well, I take a piece of cucumber every day and then have to try to get used to that taste. (Eva, a woman in her mid‐40s, symptoms for 23 months).

In addition, some participants described being able to map out combinations of foods that were manageable to eat. This exemplifies how some participants have been able to identify unique and unexpected combinations to make eating bearable. Mona had ‘found the trick’, as she put it, that ‘béarnaise sauce works … quite well for me to mask, for example, the disgusting potatoes (laughs)’. Interestingly, while potatoes were horrible to Mona, to others potatoes were a safe food, illustrating the great variation and unpredictability of the condition. Lisa further described a similar experience, expressing a fascination about how cucumber ‘with its weird taste often neutralizes the rottenness. I mean, it sounds like the worst (laughs) madness here but, but sometimes, it can be good to take, take something rotten with rotten (laughs)’. Another strategy used to increase the number of ‘safe foods’ was to occasionally revisit certain foods to assess any shifts in their taste perception. Ted describes how foods are revisited from time to time and the related emotions.

It happens sometimes, it has happened a number of times, that you (laughs) crave something very much and then (laughs), perhaps you know on some level that, like, this isn't going to be like, at all what you've imagined. You crave it as it like it used to be, that's not how it's going to be now, but, but you try anyway and then you get a bit disappointed. This has happened more than once. (Ted, a man in his late 20s, symptoms for 22 months).

In sum, adaptations to food and eating could be made in many different ways, including complete avoidance, ‘hardcore’ exposure no matter the taste, experimentation with combinations and minor testing to see if something has happened.

#### 5.2.2 Managing Olfactory Training

With the opening of the clinic in 2021, patients had the option to be referred there for targeted support. However, the participants testified to long waiting periods before getting an appointment. Initial consultations typically occurred online and were conducted by a nurse. Some participants also had the opportunity to visit the clinic in person to assess their olfactory functions, something that was appreciated. Some described the treatment offered as rather disappointing, particularly given the lengthy wait. While they appreciated receiving support and empathy for their condition, many had anticipated being presented with treatment options beyond olfactory training alone. Tina's contact with the clinic is an example:

[…] it wasn't really much help. But a little, I mean she [the nurse] was very accommodating and good in many ways, but there's, like, what you can do is this smell training then. So, there's not much more one can do, really, than that. (Tina, a woman in her late 40s, symptoms for 30 months).

A few other treatment options were, however, sometimes recommended such as nasal spray, nasal rinsing, and allergy medication, ‘not really things that are based in research or anything, but more like tips on what they have heard from other patients who have been helped and so on’, as described by Julia (a woman in her late 20s, symptoms for 16 months). At the clinic, the patients had been offered a schedule detailing which kinds of olfactory stimuli to use and how often. Some experienced improvement from the training, whereas others noticed no effect. Irrespective of perceived effects, however, it was clear from the interviews that systematic olfactory training was far from effortless. For some, it felt like a constant everyday reminder of their condition leading to feelings of sadness. Alexandra described her frustration with the training:

So it's, there's also a frustration which means that I don't always have the energy for smell training because I get sad. Because time and again I try to just remember, try to relax, try. But partly nothing happens, it feels like I've been smell training for an eternity, and nothing happens. (Alexandra, a woman in her early 30s, symptoms for 32 months).

What Alexandra means here by ‘I just try to remember’ is what a given scent smells like. When training, the instruction is to try to recall the right smell from memory while sniffing a bottle or a jar. However, this requirement to recollect was experienced as more and more difficult with time. ‘How am I supposed to tell my brain to remember something from two years ago?’, as Alexandra further expressed it. Rebecca also shared her experience about thinking of a particular scent, and how ‘it's getting harder and harder’. She elaborated further, exemplifying her increased difficulty in remembering the smell of coffee:

I can't really, I have a harder time conjuring up in my head what it's supposed to smell like, like when you smell coffee powder, for example. It has a quite strong smell to me, but it doesn't smell like coffee at all. So, but now I have, now it's hard to remember how it's really supposed to smell. (Rebecca, a woman in her early 50s, symptoms for 24 months).

While the above examples are mostly related to quantitative reductions of smell and taste, distortions added another layer of complexity to the training. In addition to difficulties in remembering, patients with parosmia also found it challenging to train with unpleasant smells. For example, ‘When it comes to garlic and things like that, instant coffee and such’, Fred (a man, in his mid‐60s, symptoms for 19 months) said, ‘it's not possible at all. It smells so bad that I couldn't keep up with the training’. Pia had similar experiences of severe discomfort during training:

I have to sit and smell this terrible mustard that makes me want to vomit when I smell it. Or whatever it is. And the ketchup, I started crying the other night because it smells so disgusting that I can't, I almost can't do this. When you just sit and retch. (Pia, a woman in her 50s, symptoms for 13 months).

Yet another dimension of olfactory training that came up was some participants feeling that they might not have done enough and, consequently, blaming themselves for the lack of improvement. Fredrik (a man in his mid‐60s, symptoms for 13 months) expressed a wish to have initiated the training earlier, ‘Because I read somewhere that the earlier you start, the better it was thought to be. And maybe put a bit more effort into it then’. The self‐blame was even clearer for Ellinor (a woman in her mid‐60s, symptoms for 23 months), saying ‘I feel a bit ashamed that I'm a bit bad at it, that I haven't done it, that I haven't tried more’. Clearly, just like the everyday managing of food and eating, engaging in systematic olfactory training demanded effort. Partly about the obvious time needed for compliance to the treatment, but also about the emotional toll of experiencing the disappearance of smells from your memory, the physical disgust when having severe parosmia and the psychological burden of blaming yourself for the perceived lack of effect. Consequently, living with the chemosensory dysfunction demanded constant emotional navigation, analysed in the last theme.

### 5.3 Navigating the Emotional Landscape

In addition to driving practical actions, the symptoms occupied much of the participants' thinking. Different strategies of thought were used to cope with the situation, illustrated in the two sub‐themes (i) self‐persuasion and suppression and (ii) the ambivalence of acceptance.

#### 5.3.1 Self‐Persuasion and Suppression

The emotional management of the dysfunction was manifested in different ways, primarily in two forms. First, participants were trying to persuade themselves that things tasted better than they actually did. Second, the situation as such was put in perspective, and relativized in comparison to something worse. Alexandra exemplifies the first form, saying ‘In my head, I have just fantasized that this is really delicious … I have kind of manipulated myself’. Similarly, Rebecca said, ‘I have convinced myself that I like coffee’. In both cases, it appears that the actual taste was unchanged. Still, they decided that from now on the taste is acceptable. As a ‘kind of manipula[tion]’, to quote Alexandra.

The second form of management was about suppressing emotions, manifested in how participants would tell themselves that there are other much worse conditions than ‘only’ having problems with smell and taste. Some tried their best to suppress their experiences by ignoring them and continuing life as usual. Jasmine suffered from feelings of being alone in her situation, rarely talking to others about it, and with no one to talk to and ‘no one who can really relate to it’. Hence,

… then I think I've mostly just kept going and not stopped to feel because I just couldn't deal with it, and I think I've often thought like, well, it could have been worse, it could have been a more serious disease. And that I can joke about it myself and be like “What a weird disease! Parosmia, like what the hell is that!?” But I'd rather have this than something else. (Jasmine, a woman in her mid‐20s, symptoms for 30 months).

Similar to Jasmine who ‘can joke about it’, Fredrik also used humour to cope:

I'm joking a bit … when people talk you're joking about how it is to have [hyposmia]… “It's great,” I say, “because pee and poo don't smell bad anymore.” It would've been good to work as a garbage collector or something. Then you wouldn't have had to suffer from the bad smells. (Fredrik, a man in his mid‐60s, symptoms for 13 months)

In addition to using humour, Fredrik was also relativising his symptoms. He was one of the few participants who did not suffer from parosmia but still had an extremely reduced sense of smell. So, he compared himself to those with parosmia:

Luckily, I don't have these cross‐connections, that things smell wrong. As some do, apparently. So, when there is a smell, it smells right. So cinnamon smells like cinnamon and mustard smells like mustard and nothing else.

Thus, one way of coping emotionally was to tell oneself that it could have been worse and to downplay the situation in contrast to ‘something else’ and ‘more serious’, but also to joke about the situation. What the two forms of emotional management presented here—persuading oneself to like something regardless of the taste and suppressing the seriousness—have in common is a form of acceptance of the new normality. If the previous themes were about actions to improve, these were accompanied by an acknowledgement that maybe it would not. However, this acceptance came with ambivalence, as seen below.

#### 5.3.2 The Ambivalence of Acceptance

It is important to stress that participants were managing and thinking about their situation differently over time. It is not about a simple timeline of initial responses followed by a certain set of changes, but rather a dynamic trajectory of ups and downs. This is especially illuminating regarding hope, which seemed particularly important early on but fluctuated over time. Hope could be difficult to keep up the longer the symptoms had been present, as Mona explains:

You've kind of clung to the hope that this will [end]. If only you had known that you're going to endure this for four years, well then, I could bear it for four years. But if you're going to think, like, that this is how it's going to be forever, then you'll go insane. (Mona, a woman in her mid‐40s, symptoms for 24 months).

The gradual loss of hope was sometimes described as a process leading more and more to an acceptance of the new situation. This trajectory is described by Penny, using the metaphor of a ‘journey’:

You could really say it's been a journey. From feeling like you've lost a lot of hope and joy in life to having learned, well, not only to get much better physically but also to learn to live with these impairments. (Penny, a woman in her mid‐20s, symptoms for 31 months).

Thus, Penny had found ways to cope, from a complete loss of hope to a new situation that still entailed impairments yet with some positivity regained. Closely related to this, some elaborated on getting to a point where they decided that they needed to change their thinking. This was partly about food and eating, for example how Pia talked about eating eggs: ‘I've gotten used to it. This is how eggs taste to me. Like, “accept it,” kind of’. She then continued, referring to her food situation in general:

I mean, it's not pleasant but it's not the same kind of horrible. In the beginning, there was so much mo‐, a lot more emotions involved or (laughs) whatever you want to call it. Now it's more just like, “Breathe. Things turned out the way they did. I just force this down and then just …” It depends a bit on what mood you're in too. Some days it's easier, other days you get reminded and sad again … (Pia, a woman in her 50s, symptoms for 13 months).

As another example, Meg explained her modified take on the situation as a whole:

I mean, I think, “I can't live like this,” that's what I think, and soon I must find some other strategies. I must, I don't know though. But, but it starts now like this, “Yes, but now I have to deal with this somehow. I have to do something different. I have to break the pattern.” … Yet, I'm a bit like, if you can't change things, then you must, you can still change the way you relate to it. So that's probably my next step. (Meg, a woman in her mid‐50s, symptoms for 36 months).

Acknowledging the situation as the new normal could also feel like giving up, which could feel like a double‐edged sword. As illustrated by Lisa, new ways of thinking and living came with a realisation that she had to accept a situation that she did not want to accept:

My thoughts go like this: I hope it will disappear. I mean, I want it to. But there's also a part of me that thinks: “Ah. Now I've had it for so long. Maybe this is just how it's going to be.” And that's why I need to learn, to accept it in life in a way that works. And I don't want to accept it too much because I also want it to disappear. So it's, it's like a balance or fight in my soul like this … if in one to two years I, of course, I hope that, that it's gone but there's almost a bigger part of me that's like, “Yes but since I've had it so long. Why would it just disappear like that?” (Lisa, a woman in her late 20s, symptoms for 29 months).

Accepting the unacceptable was a major concern for Lisa. Her ‘fight in my soul’ is a colourful metaphor for this ambivalence, of being pragmatic ‘in a way that works’ while not giving up hope that things can change for the better. Looking at several stories from the participants, it thus becomes clear that acceptance was a way of coping but also appeared to be seen as a surrender.

## 6 Discussion

In this article, we have explored trajectories of how long‐term chemosensory dysfunction after COVID‐19 is understood and managed among Swedish patients undergoing treatment for their condition, using semi‐structured interviews. It is clear from the data that the dysfunction is stressful and could lead to negative emotions, and the findings underscore the challenges faced by the participants and the significance of personal coping strategies and psychological resilience. The participants went through a phase of trying to comprehend the nature and severity of their condition and to determine their subsequent actions. Their initial evaluation varied depending whether it was early or late in the pandemic and how known these symptoms were at the time. For all, however, lack of knowledge was stressful, particularly the lack of knowledge related to what they could expect in terms of improvement, as well as the availability and effectiveness of treatments.

The process of understanding and managing the life situation that we identified among the participants has theoretical support. In line with Lazarus and Folkman's psychological stress and coping theory (Biggs, Brough, and Drummond 2017; Folkman and Lazarus 1980), participants employed various strategies after the first stage of trying to understand their symptoms: problem‐focused strategies, aimed at directly confronting the stressor or emotion‐focused, aimed at regulating the emotional response to the stressor. While this taxonomy has been criticised for not being conceptually clear, mutually exclusive or exhaustive, it provides a useful heuristic for understanding different kinds of coping (Biggs, Brough, and Drummond 2017), including making sense of our findings. What we found was that strategies used to address the stressor directly were exemplified by olfactory training (even before the onset of treatment), using corticosteroids, resorting to alternative medicine or forcing themselves to consume unpalatable food. All with the hope that this would reduce the symptoms. Additionally, instances of emotion‐focused coping were also revealed, notably through the avoidance of certain triggering foods and smells. But also attempts to endure symptoms, suppress them, relativize their severity, rely on hope, use humour or make efforts to come to terms with the situation. Beyond theory, empirical support also comes from a previous qualitative study focusing on Long COVID, showing how experiences fluctuated between uncertainty and new insights (Almgren et al. 2022). The difference compared to the present article is that taste and smell dysfunction were the main symptoms of our participants. This means that the process of initial understanding and ways of managing the conditions over time cannot straightforwardly be generalised to patients suffering from a broader palette of long COVID symptoms. The experience of brain fog, fatigue and cardiovascular manifestations is significantly different from chemosensory dysfunction.

Lazarus and Folkman's theory (Folkman and Lazarus 1980) further emphasises a dynamic process wherein individuals continually reassess (appraise) their circumstances and coping strategies, leading to emotional outcomes that can be either positive or negative. Our data illustrate this ongoing cycle, with participants adjusting their perceptions and strategies over time, especially upon recognising the prolonged nature of their symptoms. Some initially sustainable strategies, like avoidance, eventually necessitated re‐evaluation, pushing some participants to confront their conditions more directly over time. Avoidance behaviour has been documented in people with COVID‐19‐induced chemosensory dysfunction before (Burges Watson et al. 2021; Parker et al. 2021). Here, we add a longer‐term perspective to these findings, demonstrating how avoidance could be one of several coping strategies that over time might be substituted for or complemented with strategies like active exposure.

No participant confidently stated that the treatment used within healthcare, olfactory training, had positive effects (although some believed it had). In the literature, olfactory training is described as the recommended treatment for olfactory dysfunction (Gary et al. 2023; Whitcroft and Hummel 2019). It is described as a treatment that ‘carries very little risk of adverse effects, is cheap, and can be administered by the patient’ (Whitcroft and Hummel 2019). Our findings describe real‐world applications and experiences of olfactory training from the perspective of patients who have undergone this treatment. These show that the treatment could have side effects of a psychological nature, like the strain of the olfactory training, frustration over the perceived lack of results and the emotional toll of not recalling smells. In addition, living with severe parosmia could make certain odours repulsive, which could make the treatment even more arduous. Thus, while olfactory training has potential benefits, such challenges should be taken into consideration in the interaction with patients. In addition, the olfactory training was a constant reminder of their loss, and the feeling of having trained insufficiently caused guilt and self‐blame. As such, setting realistic expectations is likely important, as is the provision of psychological support, and possibly integrating other supportive therapies to enhance patient outcomes (Neuman et al. 2023). Ideally, given the personal nature of the olfactory distortions and experiences of training, healthcare professionals could aim for adopting a personalised approach, tailoring recommendations to the individual's specific situation, preferences and coping abilities. Previous studies of long COVID patients, with and without chemosensory dysfunction as a dominant symptom, have shown that both treatment and understanding of the situation have been lacking (Almgren et al. 2022; Kennelly et al. 2023; Macpherson et al. 2022; Moro‐López‐Menchero et al. 2024). Our participants, on the contrary, expressed that this emotional support and empathic approach existed at the specialised clinic. This was important even when the treatments available were perceived as disappointing, which confirms the value of meeting health professionals who show empathy and understanding for the situation, something that we have also discussed elsewhere (Neuman et al. 2024).

A key challenge in coping with chemosensory dysfunction is the uncertainty of the symptoms and the scarcity of knowledge about the prognosis. Expressions of desperation and frustration were evident in several interviews, potentially prompting the adoption of problem‐focused coping strategies that can be costly and even harmful. One participant recounted expending substantial amounts of money on alternative remedies to no avail. Another expressed a willingness to undertake almost any measure to restore her normal sense of smell. Hope and acceptance were emotion‐focused strategies used to cope with the long‐term symptoms. Leggat and colleagues note that ‘For people with Long COVID, a combination of present acceptance but hope for the future may represent their personal strategy to navigate day‐to‐day activities’ (Leggat et al. 2024, p. 13). The transition from hope for recovery to a state of acceptance was observed in the present study as well. However, our findings also demonstrate that shifting from hope to acceptance was exceedingly challenging, akin to a grieving process. Similarly, Kennelly et al. (2023) showed how patients with Long COVID grieved the past, both in terms of behaviour (what they used to be able to do) and identity (whom they used to be). As such, it is not unsurprising that acceptance could also be perceived as surrender, as some of our participants expressed since it requires a recognition that life may never return to what it once was. One can also hypothesise that ‘surrender’ might be related to the olfactory training because in some instances, acceptance among the participants seemed to include discontinuing olfactory training. If this is a common pattern in this clinical population, it is a major challenge for professionals to simultaneously communicate the uncertainties of prognosis and treatment effects without indirectly discouraging compliance.

### 6.1 Strengths and Limitations

With regard to methodological considerations of the present study, potential issues with selection bias should be highlighted. The availability of clinical services varies across different Swedish regions, potentially creating disparities in access and awareness of available treatments. Furthermore, among those informed about the study at the clinic, there might be a bias in who chooses to participate. The strengths lie in its distinctive cohort, shedding light on the daily experiences of individuals with symptoms severe enough to warrant clinical intervention, rather than solely relying on self‐reported symptoms. In addition, in comparison to other studies, we contribute with the perspective of participants having lived with the symptoms for a long period—up to 3 years. This provides insights into participants' experiences of coping with long‐term smell and taste dysfunction as well as their experiences with specialised olfactory training. It is this longer‐term perspective, in comparison with a great deal of previous research on similar populations, that allowed us to identify the fluctuating, cyclic and fluid trajectory of understanding and managing the situation, which ultimately is the main contribution of this article.

### 6.2 Recommendations for Further Research

Knowledge of the physiological mechanisms involved in long‐term chemosensory dysfunction to develop targeted treatments is crucial. Given the lack of effective treatment today, however, developing and assessing the effectiveness of cognitive‐behavioural therapy, support groups and other psychological interventions is also highly relevant.

### 6.3 Implications for Policy and Practice

Ideally, nurses should provide broad support for patients with persistent smell and taste dysfunction caused by COVID‐19, addressing both physical and psychological needs. Personalised care plans could be of value, along with patient education and expectation management. Support in olfactory training, guidance on safe practices and monitoring the use of alternative treatments are also crucial. By adopting these strategies, nurses can enhance the quality of life for affected patients, empathically guiding them in navigating the challenges of their condition while offering evidence‐based treatment.

## 7 Conclusion

This study explores the long‐term impact of COVID‐19 on smell and taste dysfunction, providing insights into the experiences of individuals navigating this condition. The trajectory from the sudden onset of smell and taste alterations to the ongoing struggle with parosmia underscores the complex nature of COVID‐19's effects beyond the acute phase of infection. Despite the availability of treatments like olfactory training, the efficacy remains varied, with many participants expressing frustration over the limited options and the (perceived) lack of significant improvement. This highlights a need for research into more effective interventions and support mechanisms. Furthermore, the study illuminates the emotional and psychological toll of smell and taste loss, with participants employing a range of coping strategies to manage their altered perceptions. The findings accentuate the necessity for a broad approach to treatment, incorporating psychological support to aid individuals in adapting to their new sensory realities. This research offers a crucial perspective on the importance of addressing the comprehensive needs of those affected by smell and taste dysfunction, emphasising the need for ongoing support, innovation in treatment options and a deeper understanding of the impacts on quality of life.

## Author Contributions

P.S., N.N., M.F., E.L. made substantial contributions to conception and design, or acquisition of data, or analysis and interpretation of data. P.S., N.N., M.F., E.L. involved in drafting the manuscript or revising it critically for important intellectual content. P.S., N.N., M.F., E.L. given final approval of the version to be published. Each author should have participated sufficiently in the work to take public responsibility for appropriate portions of the content. P.S., N.N., M.F., E.L. agreed to be accountable for all aspects of the work in ensuring that questions related to the accuracy or integrity of any part of the work are appropriately investigated and resolved.

## Ethics Statement

This study received approval from the Swedish Ethical Review Authority in May 2022 (No. 2022‐02353‐01).

## Conflicts of Interest

The authors declare no conflicts of interest.

## Peer Review

The peer review history for this article is available at https://www.webofscience.com/api/gateway/wos/peer‐review/10.1111/jan.16601.

## Supporting information


Data S1.

Data S2.

## Data Availability

The data that support the findings of this study are available from the corresponding author upon reasonable request.

## References

[jan16601-bib-0001] Almgren, J. , E. Löfström , J. S. Malmborg , J. Nygren , J. Undén , and I. Larsson . 2022. “Patients' Health Experiences of Post COVID‐19 Condition—A Qualitative Study.” International Journal of Environmental Research and Public Health 19, no. 21: 13980. 10.3390/ijerph192113980. 36360860 PMC9656359

[jan16601-bib-0002] Altundag, A. , E. Yilmaz , and M. C. Kesimli . 2022. “Modified Olfactory Training Is an Effective Treatment Method for COVID‐19 Induced Parosmia.” Laryngoscope 132, no. 7: 1433–1438. 10.1002/lary.30101. 35257391 PMC9088368

[jan16601-bib-0003] Biggs, A. , P. Brough , and S. Drummond . 2017. “Lazarus and Folkman's Psychological Stress and Coping Theory.” In The Handbook of Stress and Health: A Guide to Research and Practice, 1st ed., 349–364. New York: John Wiley & Sons, Ltd..

[jan16601-bib-0004] Braun, V. , and V. Clarke . 2006. “Using Thematic Analysis in Psychology.” Qualitative Research in Psychology 3, no. 2: 77–101. 10.1191/1478088706qp063oa.

[jan16601-bib-0005] Brinkmann, S. 2014. “Unstructured and Semi‐Structured Interviewing.” Oxford Handbook of Qualitative Research 2: 277–299.

[jan16601-bib-0006] Burges Watson, D. , M. Campbell , C. Hopkins , B. Smith , C. Kelly , and V. Deary . 2021. “Altered Smell and Taste: Anosmia, Parosmia and the Impact of Long Covid‐19.” PLoS One 16, no. 9: e0256998. 10.1371/journal.pone.0256998. 34559820 PMC8462678

[jan16601-bib-0007] Burges Watson, D. , S. Lewis , V. Bryant , et al. 2018. “Altered Eating: A Definition and Framework for Assessment and Intervention.” BMC Nutrition 4: 1–10. 10.1186/s40795-018-0221-3. 32153878 PMC7050903

[jan16601-bib-0008] Butowt, R. , K. Bilinska , and C. S. von Bartheld . 2023. “Olfactory Dysfunction in COVID‐19: New Insights Into the Underlying Mechanisms.” Trends in Neurosciences 46: 75–90. 10.1016/j.tins.2022.11.003. 36470705 PMC9666374

[jan16601-bib-0009] Croy, I. , S. Nordin , and T. Hummel . 2014. “Olfactory Disorders and Quality of Life—An Updated Review.” Chemical Senses 39, no. 3: 185–194. 10.1093/chemse/bjt072. 24429163

[jan16601-bib-0010] Folkman, S. , and R. Lazarus . 1980. “An Analysis of Coping in a Middle‐Aged Community Sample.” Journal of Health and Social Behavior 21, no. 3: 219–239. 10.2307/2136617. 7410799

[jan16601-bib-0011] Gary, J. B. , L. Gallagher , P. V. Joseph , D. Reed , D. A. Gudis , and J. B. Overdevest . 2023. “Qualitative Olfactory Dysfunction and COVID‐19: An Evidence‐Based Review With Recommendations for the Clinician.” American Journal of Rhinology & Allergy 37, no. 1: 95–101. 10.1177/19458924221120117. 35957578 PMC9379596

[jan16601-bib-0012] Helman, S. N. , J. Adler , A. Jafari , et al. 2022. “Treatment Strategies for Postviral Olfactory Dysfunction: A Systematic Review.” Allergy and Asthma Proceedings 43, no. 2: 96–105. 10.2500/aap.2022.43.210107. 35317886 PMC8984764

[jan16601-bib-0013] Hennink, M. M. , B. N. Kaiser , and V. C. Marconi . 2017. “Code Saturation Versus Meaning Saturation: How Many Interviews Are Enough?” Qualitative Health Research 27, no. 4: 591–608. 10.1177/1049732316665344. 27670770 PMC9359070

[jan16601-bib-0014] ICT Services and System Development and Division of Epidemiology and Global Health . 2015. “Open Code 4.03 [Computer Software]. Umeå University.” https://www.umu.se/en/department‐of‐epidemiology‐and‐global‐health/research/open‐code2/.

[jan16601-bib-0015] Kennelly, C. E. , A. T. P. Nguyen , N. Y. Sheikhan , et al. 2023. “The Lived Experience of Long COVID: A Qualitative Study of Mental Health, Quality of Life, and Coping.” PLoS One 18, no. 10: e0292630. 10.1371/journal.pone.0292630. 37831706 PMC10575511

[jan16601-bib-0040] Leggat, F. J. , C. Heaton‐Shrestha , J. Fish , et al. 2024. “An Exploration of the Experiences and Self‐Generated Strategies Used When Navigating Everyday Life With Long Covid.” BMC Public Health 24: 789. 10.1186/s12889-024-18267-6. 38481230 PMC10938753

[jan16601-bib-0016] Liu, D. T. , M. Sabha , M. Damm , et al. 2021. “Parosmia Is Associated With Relevant Olfactory Recovery After Olfactory Training.” Laryngoscope 131, no. 3: 618–623. 10.1002/lary.29277. 33210732

[jan16601-bib-0017] Liu, Z. Y. , L. A. Vaira , P. Boscolo‐Rizzo , A. Walker , and C. Hopkins . 2023. “Post‐Viral Olfactory Loss and Parosmia.” BMJ Medicine 2, no. 1: e000382. 10.1136/bmjmed-2022-000382. 37841969 PMC10568123

[jan16601-bib-0018] Llana, T. , M. Mendez , S. Garces‐Arilla , V. Hidalgo , M. Mendez‐Lopez , and M.‐C. Juan . 2023. “Association Between Olfactory Dysfunction and Mood Disturbances With Objective and Subjective Cognitive Deficits in Long‐COVID.” Frontiers in Psychology 14: 1076743. 10.3389/fpsyg.2023.1076743. 36818111 PMC9932904

[jan16601-bib-0019] Macpherson, K. , K. Cooper , J. Harbour , D. Mahal , C. Miller , and M. Nairn . 2022. “Experiences of Living With Long COVID and of Accessing Healthcare Services: A Qualitative Systematic Review.” BMJ Open 12, no. 1: e050979. 10.1136/bmjopen-2021-050979. PMC8753091 35017239

[jan16601-bib-0020] Maxwell, J. A. 2024. “Collecting Qualitative Data: A Realist Approach.” In The SAGE Handbook of Qualitative Data Collection, 19–31. London, UK: SAGE Publications Ltd. 10.4135/9781526416070.

[jan16601-bib-0021] Melanie, D. , S. Zara , H. Nora , and H. Claire . 2024. “Recovery Rates and Long‐Term Olfactory Dysfunction Following COVID‐19 Infection.” World Journal of Otorhinolaryngology—Head and Neck Surgery 10, no. 2: 121–128. 10.1002/wjo2.163. 38855291 PMC11156684

[jan16601-bib-0022] Moro‐López‐Menchero, P. , M. B. Martín‐Sanz , C. Fernandez‐de‐las‐Peñas , et al. 2024. “Living and Coping With Olfactory and Taste Disorders: A Qualitative Study of People With Long‐COVID‐19.” Healthcare 12, no. 7: 754. 10.3390/healthcare12070754. 38610176 PMC11011467

[jan16601-bib-0023] Neuman, N. , E. Lövestam , J. Karlén , and P. Sandvik . 2024. “Sensing Sociality: Disruptions of Social Life When Living With Chemosensory Dysfunctions After COVID‐19.” Qualitative Health Research. Published ahead of print, October 10, 2024. 10.1177/10497323241278551. PMC12056266 39388619

[jan16601-bib-0024] Neuman, N. , P. Sandvik , N. B. Lindholm , K. Bömer‐Schulte , and E. Lövestam . 2023. “Food‐Related Experiences and Behavioral Responses Among People Affected by Chemosensory Dysfunctions Following COVID‐19: A Scoping Review.” Research in Nursing & Health 46, no. 4: 385–399. 10.1002/nur.22315. 37171788

[jan16601-bib-0025] Nowell, L. S. , J. M. Norris , D. E. White , and N. J. Moules . 2017. “Thematic Analysis: Striving to Meet the Trustworthiness Criteria.” International Journal of Qualitative Methods 16, no. 1: 1609406917733847. 10.1177/16094069177338.

[jan16601-bib-0026] O'Brien, B. C. , I. B. Harris , T. J. Beckman , D. A. Reed , and D. A. Cook . 2014. “Standards for Reporting Qualitative Research: A Synthesis of Recommendations.” Academic Medicine 89, no. 9: 1245–1251. https://journals.lww.com/academicmedicine/fulltext/2014/09000/standards_for_reporting_qualitative_research__a.21.aspx. 24979285 10.1097/ACM.0000000000000388

[jan16601-bib-0027] Parker, J. K. , C. E. Kelly , B. C. Smith , A. F. Kirkwood , C. Hopkins , and S. Gane . 2021. “Patients' Perspectives on Qualitative Olfactory Dysfunction: Thematic Analysis of Social Media Posts.” JMIR Formative Research 5, no. 12: e29086. 10.2196/29086. 34904953 PMC8673716

[jan16601-bib-0028] Parker, J. K. , L. Methven , R. Pellegrino , B. C. Smith , S. Gane , and C. E. Kelly . 2022. “Emerging Pattern of Post‐COVID‐19 Parosmia and Its Effect on Food Perception.” Food 11, no. 7: 967. 10.3390/foods11070967. PMC8997629 35407054

[jan16601-bib-0029] Parma, V. , K. Ohla , M. G. Veldhuizen , et al. 2020. “More Than Smell—COVID‐19 Is Associated With Severe Impairment of Smell, Taste, and Chemesthesis.” Chemical Senses 45, no. 7: 609–622. 10.1093/chemse/bjaa041. 32564071 PMC7337664

[jan16601-bib-0030] Pieniak, M. , A. Oleszkiewicz , V. Avaro , F. Calegari , and T. Hummel . 2022. “Olfactory Training—Thirteen Years of Research Reviewed.” Neuroscience & Biobehavioral Reviews 141: 104853. 10.1016/j.neubiorev.2022.104853. 36064146

[jan16601-bib-0031] Porter, S. 2007. “Validity, Trustworthiness and Rigour: Reasserting Realism in Qualitative Research.” Journal of Advanced Nursing 60, no. 1: 79–86. 10.1111/j.1365-2648.2007.04360.x. 17824942

[jan16601-bib-0032] Sandelowski, M. 2000. “Whatever Happened to Qualitative Description?” Research in Nursing & Health 23, no. 4: 334–340. 10.1002/1098-240X(200008)23:4<334::AID-NUR9>3.0.CO;2-G. 10940958

[jan16601-bib-0033] Sandelowski, M. 2010. “What's in a Name? Qualitative Description Revisited.” Research in Nursing & Health 33, no. 1: 77–84. 10.1002/nur.20362. 20014004

[jan16601-bib-0034] Santos, R. E. A. , M. G. da Silva , M. C. B. do Monte Silva , et al. 2021. “Onset and Duration of Symptoms of Loss of Smell/Taste in Patients With COVID‐19: A Systematic Review.” American Journal of Otolaryngology 42, no. 2: 102889. 10.1016/j.amjoto.2020.102889. 33445036 PMC7833280

[jan16601-bib-0035] Spielman, A. I. 1998. “Chemosensory Function and Dysfunction.” Critical Reviews in Oral Biology & Medicine 9, no. 3: 267–291. 10.1177/10454411980090030201. 9715366

[jan16601-bib-0036] Tan, B. K. J. , R. Han , J. J. Zhao , et al. 2022. “Prognosis and Persistence of Smell and Taste Dysfunction in Patients With Covid‐19: Meta‐Analysis With Parametric Cure Modelling of Recovery Curves.” BMJ 378: e069503. 10.1136/bmj-2021-069503. 35896188 PMC9326326

[jan16601-bib-0037] Vargas‐Gandica, J. , D. Winter , R. Schnippe , et al. 2020. “Ageusia and Anosmia, a Common Sign of COVID‐19? A Case Series From Four Countries.” Journal of Neurovirology 26, no. 5: 785–789. 10.1007/s13365-020-00875-8. 32666422 PMC7359421

[jan16601-bib-0038] Whitcroft, K. L. , and T. Hummel . 2019. “Clinical Diagnosis and Current Management Strategies for Olfactory Dysfunction: A Review.” JAMA Otolaryngology. Head & Neck Surgery 145, no. 9: 846–853. 10.1001/jamaoto.2019.1728. 31318413

[jan16601-bib-0039] Winter, A. L. , S. Henecke , J. N. Lundström , and E. Thunell . 2023. “Impairment of Quality of Life due to COVID‐19‐Induced Long‐Term Olfactory Dysfunction.” Frontiers in Psychology 14: 1165911. 10.3389/fpsyg.2023.1165911. 37151341 PMC10157159

