# Supplementary material for: Trajectories of Coping With Persistent Smell and Taste Dysfunction After a Covid‐19 Infection—A Qualitative Interview Study
Source: J Adv Nurs. 2024 Nov 7;81(7):4085–97. doi: 10.1111/jan.16601 (PMC12159357; doi:10.1111/jan.16601)
Supplement: Supplementary file 1 — Data S1. [file JAN-81-4085-s001.docx]

INTERVIEW GUIDE – translated from Swedish to English only for the purpose of supplementing the paper Trajectories of coping with persistent smell and taste dysfunction after a Covid-19 infection - a qualitative interview study.

PART 1 – SYMPTOMS

- Can you tell me what led you to seek help at the clinic?
- What help have you received?
  - How did you experience the wait for help?
  - Is there anything you feel you missed out on, something you would have liked help with?
- Would you like to describe how your symptoms are manifesting today?
- Has it been like this throughout the entire process (since you first started experiencing the symptoms)?
  - Depending on the response, potential follow-up questions. Especially if they noticed any changes after, for example, smell training.
- Can you describe how you reacted when you first experienced these symptoms?
  - How did you handle it?
  - Have you used any specific strategies? Mention meals if it doesn’t come up.
- After it had been going on for a while, did you continue to handle it the same way, or did it change?
  - Again: specific strategies, and specifically ask about meals if it doesn’t come up.
- How do you feel your surroundings have handled your situation?
  - Family, co-workers, friends, etc.
- Where have you been able to find support? (For example, family, friends, healthcare, the internet, patient associations)

**PART 2 – DAILY LIFE**

- Can you describe what a typical day currently looks like for you in terms of food and drink? A completely average weekday and an average weekend day.
- Is there anything special that you now eat on special occasions?
- Was it the same before the symptoms?
  - Depending on the response, ask follow-up questions. For example, if the participant just says it was different—"Can you tell me more about that?" "Is there anything you completely avoid now?"
- How is the responsibility for food managed in the household? That is, who mainly plans meals, does the shopping, cooks, washes dishes, etc.?
- What were these things like before you got sick? Both in terms of what you eat and drink and how the work around food was handled.
- Are there any situations or occasions you feel are particularly affected?
  - (Such things may have already come up naturally in the previous points. If so, start the question with "You’ve already touched on this, but are there any...?").
  - If not mentioned spontaneously, try to specifically find out if there’s anything related to social situations and at work.
  - Are there situations you now completely avoid?
- Has this period affected your daily life in other ways, socially? Things not directly related to eating.
  - The participant might wonder what we mean, and in that case, we can give examples like whether food costs have changed, if they’ve developed new interests, new ways of socializing, etc.
- Do you feel this period has affected your health? Physically, mentally, and emotionally.
  - If not mentioned, ask if the participant feels that the healthiness of their eating habits has been affected.
  - Anything Covid-specific? For example, reading so much that it became mentally exhausting, etc.

**PART 3 – THE FUTURE**

- Does your current situation affect how you think about the future?
  - When you think ahead, do you believe you will still have the symptoms in, say, one or two years?
- What kind of help do you think you will need from healthcare in the future?
