# Supplementary material for: Trajectories of Coping With Persistent Smell and Taste Dysfunction After a Covid‐19 Infection—A Qualitative Interview Study
Source: J Adv Nurs. 2024 Nov 7;81(7):4085–97. doi: 10.1111/jan.16601 (PMC12159357; doi:10.1111/jan.16601)
Supplement: Supplementary file 2 — Data S2. [file JAN-81-4085-s002.docx]

**Background questionnaire:** translated from Swedish to English only for the purpose of supplementing the paper Trajectories of coping with persistent smell and taste dysfunction after a Covid-19 infection - a qualitative interview study.

**Gender**

- ☐ Man
- ☐ Woman
- ☐ I do not identify as either man or woman
- ☐ Prefer not to say

**Age**

Please specify your year of birth: ______

**What is your highest completed level of education?**

- ☐ Primary school, elementary school, middle school, or similar
- ☐ 2-year high school or vocational school
- ☐ 3-4 year high school
- ☐ Folk high school or similar
- ☐ University or college, less than 3 years
- ☐ University or college, 3 years or longer

**What is your current occupation? Multiple options can be selected.**

- ☐ Employed. Specify % of full-time: **working part-time**_______
- ☐ Self-employed
- ☐ On leave or parental leave
- ☐ Studying, interning
- ☐ Participating in a labour market program
- ☐ Unemployed
- ☐ Retired
- ☐ Receiving sickness or activity compensation (early retirement or disability pension)
- ☐ Long-term sick leave (more than 3 months)
- ☐ Managing own household
- ☐ Other

**Who do you live with? That is, who do you live with at least half of the time? Multiple options can be selected.**

- ☐ No one
- ☐ Parents or siblings
- ☐ Spouse, partner, or significant other
- ☐ Other adults
- ☐ Children

**If there are children in the household: How many? Multiple options can be selected.**

- ☐ 0-5 years old. Specify number here: __________________________
- ☐ 6-12 years old. Specify number here: ________________________
- ☐ 13-17 years old. Specify number here: ____________________
- ☐ 18 years or older. Specify number here: _________________

**When did you first notice that your sense of taste and/or smell was affected?** Specify month and year:________________

**Since when have you been in contact with the Smell and Taste Clinic?** Specify month and year: ______

**At the onset of symptoms, which of the following describes your symptoms? Multiple options can be selected.**

- ☐ Sense of smell completely or almost completely gone
- ☐ Sense of smell partially reduced
- ☐ Altered sense of smell (i.e., things smell different rather than just less)
- ☐ I have not experienced any affected sense of smell
- ☐ Sense of taste completely or almost completely gone
- ☐ Sense of taste partially reduced
- ☐ Altered sense of taste (i.e., things taste different rather than just less)
- ☐ I have not experienced any affected sense of taste

**Today, which of the following describes your symptoms? Multiple options can be selected.**

- ☐ Sense of smell completely or almost completely gone
- ☐ Sense of smell partially reduced
- ☐ Altered sense of smell (i.e., things smell different rather than just less)
- ☐ I have not experienced any affected sense of smell
- ☐ Sense of taste completely or almost completely gone
- ☐ Sense of taste partially reduced
- ☐ Altered sense of taste (i.e., things taste different rather than just less)
- ☐ I have not experienced any affected sense of taste
